# Supplementary material for: Novel Esomeprazole Magnesium-Loaded Dual-Release Mini-Tablet Polycap: Formulation, Optimization, Characterization, and In Vivo Evaluation in Beagle Dogs
Source: Pharmaceutics. 2022 Jul 5;14(7):1411. doi: 10.3390/pharmaceutics14071411 (PMC9323828; doi:10.3390/pharmaceutics14071411)
Supplement: Supplementary file 1 [file pharmaceutics-14-01411-s001.zip › pharmaceutics-1771959-supplementary.pdf]

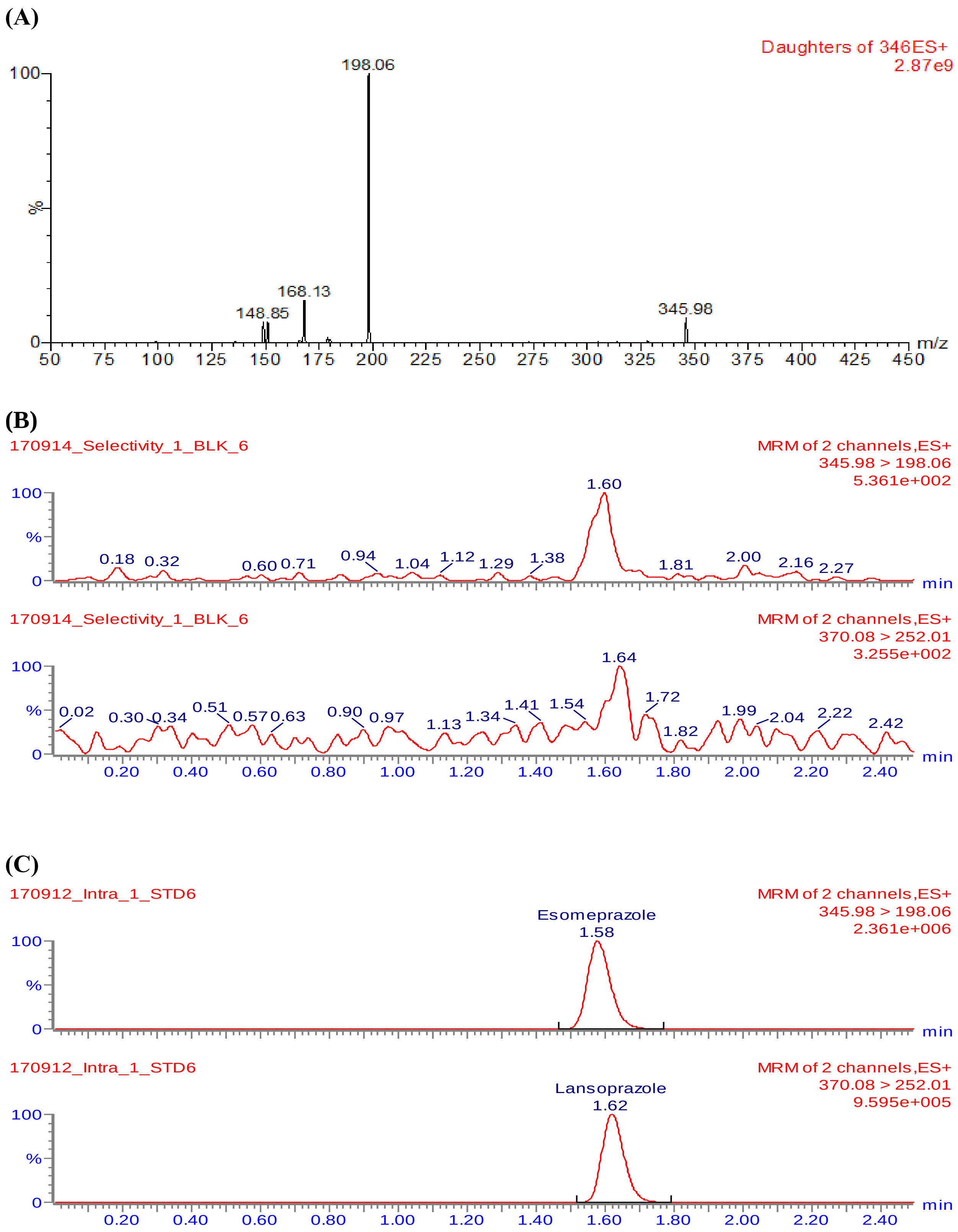

Figure S1. (**A**) MS/MS spectrum of Esomeprazole. (Esomeprazole with monitoring at m/z 346 → 198). (**B**) LC-MS/MS chromatogram of blank Beagle dogs' plasma. (**C**) LC-MS/MS chromatogram of Esomeprazole in standard plasma (500 ng/mL).

Table S1. Calibration curve validation data for Esomeprazole (n = 4)

	Nominal conc. (ng/mL)	Calculated Conc (ng/mL)				Mean	Accuracy of Mean (%)	Precision of mean (C.V., %)
		Intra	Inter 1	Inter 2	Inter 3			
STD1	1	1.019	1.022	0.997	1.021	1.015	101.5	1.2
STD2	5	4.649	4.564	5.365	4.806	4.846	96.9	7.4
STD3	10	9.495	9.532	8.998	8.877	9.226	92.3	3.6
STD4	50	47.751	47.499	46.736	43.800	46.447	92.9	3.9
STD5	100	103.112	106.676	99.940	99.796	102.381	102.4	3.2
STD6	500	526.607	537.301	524.601	531.591	530.025	106.0	1.1
STD7	1000	1055.594	1036.810	1040.007	1100.732	1058.286	105.8	2.8
STD8	5000	5030.745	4917.945	5036.044	5459.656	5111.098	102.2	4.7

Table S2. Accuracy & precision validation data for Esomeprazole (Intra: n = 5, Inter: n = 4)

Nominal conc. (ng/mL)	Accuracy (%)		Precision (C.V., %)	
	Intra	Inter	Intra	Inter
1	107.5	109.2	10.1	1.7
3	97.9	106.6	3.9	6.4
800	106.1	107.6	0.8	3.6
4000	105.4	105.1	1.3	1.8

Table S3. Recovery validation data for Esomeprazole (n = 3).

Number	Esomeprazole						Lansoprazole	
	Low (3 ng/mL)		Medium (800 ng/mL)		High (4000 ng/mL)		ISTD (2000 ng/mL)	
	A	B	A	B	A	B	C	D
1	0.015	0.012	4.543	3.694	20.868	17.178	0.188	0.166
2	0.014	0.012	4.468	3.476	20.497	17.049	0.188	0.168
3	0.014	0.012	4.431	3.535	20.848	17.144	0.182	0.166
Mean	0.015	0.012	4.480	3.568	20.738	17.123	0.186	0.167
C.V (%)	0.01	0.01	4.46	3.53	20.69	17.11	0.19	0.17
Mean recovery (%)	**82.0**		**79.6**		**82.6**		**89.6**	

A: **Unextracted STD peak area** / Unextracted ISTD peak area

B: **Extracted STD peak area** / Unextracted ISTD peak area

C: **Unextracted ISTD peak area** / Unextracted STD peak area

D: **Extracted ISTD peak area** / Unextracted STD peak area
